# 3D Printed Ion-Selective Electrodes Enriched with ZnO Nanoparticles for Potassium Detection

**DOI:** 10.3390/s26061960

**Published:** 2026-03-20

**Authors:** Ita Hajdin, Ante Prkić

**Affiliations:** Faculty of Chemistry and Technology, University of Split, Ruđera Boškovića 35, 21000 Split, Croatia; ihajdin@ktf-split.hr

**Keywords:** ion-selective electrode, 3D print, potassium determination, ZnO NPs, potentiometry, SEM/EDS, XRF, ATR-FTIR

## Abstract

Ion-selective electrodes (ISEs) are widely used analytical tools for the determination of specific ions in a variety of analytical applications due to their simplicity, selectivity, and low cost. Recent developments in materials science and digital fabrication have opened new opportunities for redesigning ISEs using modern manufacturing techniques. Here, we present a new application of 3D printing for fabricating potassium-selective electrodes using a simplified membrane composition. The 3D printing cocktail was prepared by mixing potassium tetraphenylborate, silver sulfide or graphite, and industrial ABS (acrylonitrile Butadiene Styrene) polymer. Membranes were tested both without and with the addition of ZnO nanoparticles. Incorporation of ZnO NPs significantly enhanced the electrode slope, while graphite-based membranes exhibited faster response, with potential stabilizing within 3–7 s across a concentration range of 4.88 × 10^−5^ mol L^−1^ to 1.00 × 10^−2^ mol L^−1^. The optimized 3D printed membrane containing 0.6% ZnO NPs showed near-Nernstian behaviour (slope: 59.178 mV per decade and *R*^2^ = 0.9989), a limit of detection of 2.06 × 10^−5^ mol L^−1^ and high selectivity against common interfering ions. These results demonstrate that 3D printing combined with a suitable membrane composition and nanoparticle incorporation provides a versatile platform for rapid, reproducible, and high-performance potassium ISEs.

## 1. Introduction

Potassium is an important electrolyte that plays a central role in cellular signalling, neuromuscular function, and cardiovascular stability, making its precise regulation essential for maintaining physiological balance [1,2]. Even the smallest deviations in potassium levels can have significant clinical consequences: elevated concentrations signal conditions such as acute or chronic renal impairment, hypoaldosteronism, and rhabdomyolysis, and can rapidly progress to severe cardiac arrhythmias [3,4]. Conversely, potassium deficiency is associated with muscle dysfunction, metabolic disturbances, and heightened risk of cardiovascular illnesses [5,6]. Despite the clinical relevance of electrolyte assessments, routine measurements remain largely confined to centralized laboratories equipped with complex analytical instruments and operated by trained personnel. Such constrained infrastructures limit accessibility, responsiveness, and the possibility of continuous or preventive monitoring—an issue of increasing importance given the global rise in chronic kidney disease, hypertension, and metabolic disorders [7,8]. This growing demand for decentralized ways of measuring electrolytes highlights the importance of developing robust miniature technologies capable of selective potassium detection in complex biological matrices.

Ion-selective electrodes (ISEs) are widely employed electrochemical sensors due to their high selectivity, sensitivity, and operational simplicity across biomedical, environmental, and industrial applications [9,10]. Among them, polymeric membrane ISEs incorporating ionophores have shown significant success in clinical settings, enabling accurate measurements of electrolytes in fluids such as blood, serum, urine, and sweat. Advances in ionophore design, ion-to-electron transducing layers, and miniaturized reference electrodes have further enhanced sensor performance and versatility [11,12]. Compared to conventional analytical methods, ISEs provide a rapid, cost-effective, and portable approach for ion quantification without requiring sample pretreatment [13].

Printing technologies have become central to the advancement of modern electrochemical sensors, offering flexible, precise, and scalable approaches for electrode and device fabrication [14,15]. Among these, 3D printing remains one of the most reliable and widely applied methods, particularly suited for producing ion-selective electrodes (ISEs) [16]. Owing to its simplicity, reproducibility, and compatibility with various functional inks and substrates, 3D printing enables rapid and cost-effective fabrication of miniaturized ISEs with well-defined geometry, controlled film thickness, and good mechanical stability. This technique allows direct integration of ion-selective membranes, reference layers, and conductive tracks on compact platforms, which is highly advantageous for portable and disposable electrochemical sensors [17,18]. In parallel, emerging digital manufacturing techniques, such as inkjet and 3D printing, have introduced new levels of design freedom and structural control. Inkjet printing provides precise, contact-free deposition suitable for thin, planar films, whereas 3D printing or additive manufacturing enables the creation of volumetric, customized architectures [19]. Building on these fabrication capabilities, recent advances in printable materials have enabled the integration of ion-selective components directly into conductive pastes, allowing for precise control over membrane composition, thickness, and spatial arrangement. This approach enables the fabrication of electrodes for specific target ions while maintaining the mechanical robustness and reproducibility of printed devices [20]. Additionally, the combination of multiple functional layers in a single deposition step allows for compact, multi-analyte sensor configurations, enhancing device versatility [21]. Ongoing research is also exploring hybrid strategies, where traditional printed layers are complemented by microstructured supports or patterned features created through additive manufacturing, further expanding the design possibilities for next-generation ion-selective electrodes [22]. Additive manufacturing has been successfully applied to the fabrication of solid-contact ISEs for the determination of various metal ions, including potassium, sodium, calcium, and heavy metals, demonstrating the versatility of 3D printing for producing customized sensor geometries. These approaches often combine printed electrode bodies or conductive substrates with conventional ion-selective membranes and solid-contact transducers, highlighting the potential for miniaturized and application-specific potentiometric sensors [23,24,25,26].

Recently, numerous potassium-selective ISEs have been developed across a variety of sensing platforms, including 3D-printed flow systems [27], wearable devices [5] and point-of-care sensors [8,19,20], automated fabrication setups [13], microfluidic thermoplastic arrays [8] and other miniaturised formats [28]. Despite these differing architectures, their chemical design is notably consistent: most rely on polymeric ion-selective membranes containing valinomycin as the potassium ionophore, combined with solid-contact layers based on conductive polymers such as poly(3,4-ethylenedioxythiophene) (PEDOT) or poly(3-octylthiophene-2,5-diyl) (POT) [5], carbon-nanomaterial transducers such as octadecylamine-functionalized multi-walled carbon nanotubes (OD-MWCNT) [29], or graphite-derived composites [8]. In recent years, composite materials have attracted considerable attention as solid-contact layers in ISEs, as they combine conductive matrices with materials exhibiting high capacitance or favourable interfacial properties. Such hybrid systems can enhance ion-to-electron transduction, improve potential stability, and reduce the likelihood of water layer formation at the electrode interface [30]. These recurring material choices provide stable potentiometric behaviour and allow comparable electrode performance across otherwise heterogeneous device configurations, including those produced using 3D-printing technologies.

In this study we report on the first fabrication of 3D printed solid-contact ISEs for potassium ion determination using KB(Ph)_4_ as an ion exchanger, industrial ABS as a plasticizer, graphite or Ag_2_S as charge transmitters and ZnO NPs for improving charge transfer and slope stabilization. Industrial ABS was selected due to its compatibility with the 3D-printing setup used in this work, enabling straightforward fabrication of the membrane components. Ag_2_S was included as a charge-transducing material based on its successful use in our previous sensor designs, while graphite was investigated as an alternative transducer because of its good electrical conductivity, low cost, and chemical stability, as well as the absence of photosensitivity associated with some silver-based materials. The 3D printed membranes were integrated into the miniaturized electrode body as described in our earlier publications [31,32]. The performance of our newly developed sensor was investigated, and the electrodes showed excellent sensitivity to potassium, selectivity for common interferences, and excellent reproducibility. Overall, the electrode design and fabrication presented in this study represent a fully laboratory-fabricated potentiometric sensor offering a practical, fast, and reliable solution for potassium ion quantification.

## 2. Materials and Methods

All chemicals for membrane preparation are commercially available. Potassium tetraphenylborate (KB(Ph)_4_, 97%, Thermo Fisher Scientific, Waltham, MA, USA), graphite (WWR chemicals, Radnor, PA, USA), silver sulfide (Ag_2_S, 99%, Haverhill, MA, USA), industrial ABS (Harz Labs, Moscow, Russia) and zinc oxide nanoparticles (ZnO, Haverhill, MA, USA) were used in specific mass ratios for the membrane construction. Sodium nitrate (NaNO_3_, p.a.), ammonium chloride (NH_4_Cl, p.a.), calcium nitrate tetrahydrate (Ca(NO_3_)_2_ × 4H_2_O p.a.), magnesium nitrate hexahydrate (Mg(NO_3_)_2_ × 6H_2_O p.a.), were obtained from Kemika (Zagreb, Croatia) and employed in the determination of selectivity coefficients. All potentiometric measurements were performed using a double-junction silver/silver chloride reference electrode (Ag/AgCl; Reference Plus, Mettler Toledo, Columbus, OH, USA) in conjunction with the custom-fabricated ion-selective electrodes. The electrode potentials were recorded with a SevenExcellence millivoltmeter (Mettler Toledo, Columbus, OH, USA) interfaced with a PC running LabX Direct pH (version 3.3, Mettler-Toledo, Greifensee, Switzerland/Columbus, OH, USA) software.

Scanning electron microscopy (SEM) was performed using a field-emission scanning electron microscope (JSM-7610F Plus, JEOL, Tokyo, Japan). Prior to imaging, membrane samples were sputter-coated with a 5 nm thick platinum layer and mounted on aluminum stubs. SEM micrographs were acquired at magnifications of 1000×, 10,000×, 20,000× and 50,000×. Energy-dispersive X-ray spectroscopy (EDS), integrated into the SEM system (JSM-7610F Plus, JEOL, Japan), was used for elemental analysis of the membranes. EDS spectra and elemental maps were collected at a magnification of 1000×.

X-ray fluorescence (XRF) analysis was performed using a Bruker ARTAX spectrometer (Mo anode, 50 kV, 400 µA, live time 60 s, air atmosphere) to verify the elemental composition of membranes. Spectra were evaluated using a standard Bayesian method.

Attenuated total reflectance Fourier transform infrared (ATR-FTIR) spectroscopy was performed using an Alpha II spectrometer (Bruker Optik GmbH, Ettlingen, Germany) equipped with a Platinum ATR module. Spectra were recorded in the 4000–400 cm^−1^ range with a resolution of 4 cm^−1^ using 24 scans for both sample and background measurements.

### 2.1. Membranes Preparations

**Figure 1:**
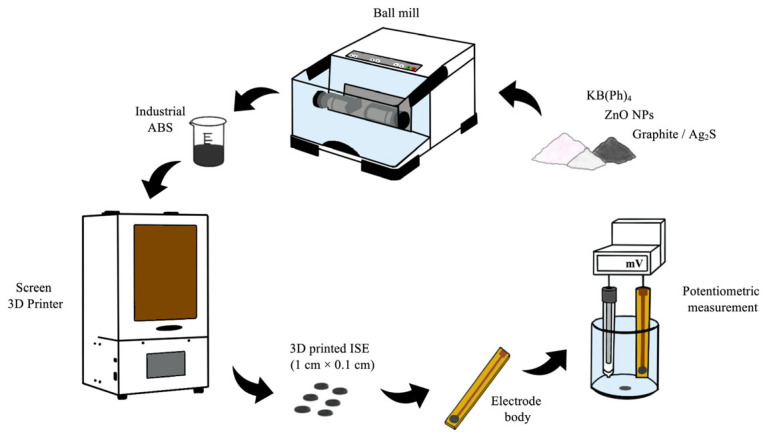
Preparation of the 3D printed ISE for potassium ion determination.

Membranes were printed using Phrozen Sonic 4k 3D printer (Phrozen, New Taipei City, Taiwan). Draft models of the membranes were created in Sketchup software (version 2023, Trimble Inc., Sunnyvale, CA, USA), while ctg files were created in Chitubox application (version 1.9.3, CBD-Tech, Shenzhen, China). The printing mixture was obtained by homogenizing all the powder compounds (KB(Ph)_4_, graphite or silver sulfide and ZnO NPs) all together in ball mill for 3 min and combining it with industrial ABS resin afterwards. Different membrane compositions were tested in order to obtain the best slope and factor of correlation (*R*^2^). The final concentrations of KB(Ph)_4_ (3%), graphite (1%), Ag_2_S (2%), industrial ABS (94–96%), and ZnO nanoparticles (0.1–0.6%) were selected to balance two factors: achieving satisfactory potentiometric response and ensuring proper 3D printability. Higher fractions of powdered components hindered printing, while lower fractions compromised sensor performance, so the chosen ratios allowed effective fabrication with minimal chemical consumption. ZnO nanoparticles were incorporated at an optimal concentration of 0.6% to enhance the electrode slope. They increase the effective surface area at the membrane-electrode interface, facilitating more efficient charge transfer between the ion-selective membrane and the underlying solid-contact layer. Furthermore, ZnO helps stabilize the electrode potential by minimizing the formation of an interfacial water layer, thereby reducing potential drift and improving long-term reproducibility of the potentiometric response. All membranes were printed with a diameter of 1 cm and a thickness of 0.1 cm. Six membranes were printed at the same time using identical printing parameters. After printing, the membranes were cleaned using isopropyl alcohol and cured for 15 min in UV chamber. Conductive graphite adhesive was used to attach the printed membrane to the electrode body. After drying for 24 h, the copper layer and the interface between the membrane and the body were insulated with a non-conductive glaze to prevent any interaction with the test solution. The complete process is shown in Figure 1. The surfaces of the printed membranes were polished with 1000, 2000 and 3000 grit size sandpaper after each measurement to remove any deposits that may have formed during the previous measurement. The compositions of all 3D printed membranes are presented in Table 1.

### 2.2. Membrane Testing

The membranes were tested in 0.1 M solution of potassium nitrate (KNO_3_, p.a., Kemika, Zagreb, Croatia). The solution used to test the membranes were prepared by standard dilution method in ultrapure water with a declared conductivity of 0.04 μS cm^−1^ (Millipore Simplicity, Billerica, MA, USA) at 25 °C. Each membrane was tested five times, confirming consistent and reliable electrode responses. After each measurement, the electrodes were rinsed with Milli-Q water, gently dried with paper tissue, and stored in a vacuum chamber until further use.

In KNO_3_ solution, K^+^ ions diffuse to the composite membrane and interact primarily through ion-pair formation with BPh_4_^−^, while additional electrostatic interactions occur at the ZnO surface. Graphite serves as a conductive pathway, facilitating electron transfer and enabling potentiometric signal generation.

To optimize the electrode response to K^+^, we systematically adjusted multiple parameters and evaluated the performance of the K^+^ ISEs by determining the limit of detection (LOD), the limit of quantification (LOQ), and the slope of the electrode response. For monovalent cations, the Nernst Equation (1) predicts a theoretical slope of 59.2 mV per decade, as it relates the standard potential (*E*^0^, mV), ionic charge (*z*), and analyte concentration (*c*).

(1) $$E=E^{0}+\frac{59.2\;mV}{z}\;\log\left(c\right)$$

### 2.3. Selectivity Coefficients

The selectively of the K^+^ ISE was evaluated using the fixed interference method, following standard potentiometric procedures [33,34]. The electrode was first conditioned overnight in a 0.1 M KNO_3_ solution to ensure stable baseline potential. EMF measurements were subsequently performed in the presence of common interfering cations, including sodium (0.1 M NaNO_3_), ammonium (0.1 M NH_4_Cl), calcium (0.1 M Ca(NO_3_)_2_ × 4H_2_O), and magnesium (0.1 M Mg(NO_3_)_2_ × 6H_2_O). To prevent cross-contamination, the electrode and the measurement vessel were thoroughly rinsed with ultrapure water and dried between measurements. Selectivity coefficients (log *K*^pot^) for each interferent were then calculated according to Equation (2), providing a quantitative assessment of the electrode’s ability to discriminate K^+^ over potential interferents under well-defined conditions. It should be noted that Equation (2) uses concentrations (*C_a_* and *C_x_*) rather than activities. Since the measurements were performed at relatively high ionic strength (0.1 M of interfering ions), the resulting selectivity coefficients are apparent values and should be interpreted accordingly.

(2) $$\log K_{a,x}^{pot}=\frac{{Z_{a}\;(E}_{x}-E_{a})}{59.2}+\log\left(\frac{C_{a}}{C_{x}^{Z_{a}/Z_{x}}}\right)$$

Here, *K*^pot^ is the selectivity coefficient, *Z*_a_ and *Z*_χ_ are the charges of the analyte and interfering ion, *E*_a_ and *E*_χ_ are their corresponding emf responses while *C*_a_ and *C*_χ_ are their respective concentrations.

## 3. Results and Discussion

### 3.1. Membrane Testing

**Figure 2:**
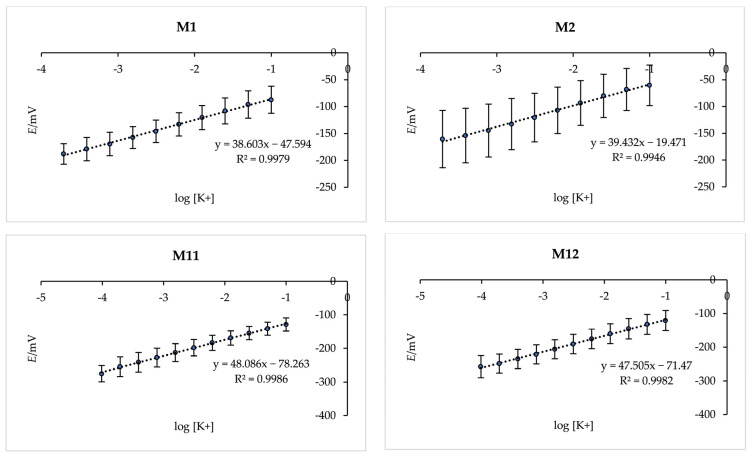

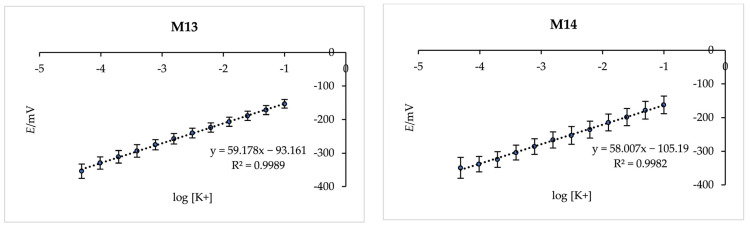
3D printed K^+^ ISEs (**M1**, **M2**, **M11**, **M12**, **M13** and **M14**) with different compositions and their responses to potassium ions. Error bars here represent standard deviations obtained from five repeated measurements of the same membrane (*n* = 5).

To evaluate the effect of membrane composition on the potentiometric response of the K^+^ ISE, several membrane examples were 3D printed and systematically compared. The set included membranes without ZnO NPs (**M1** and **M2**) as well as those containing 0.1–0.6% (*w*/*w*) of ZnO NPs (**M3**–**M14**) as presented in Table 1. The membranes without ZnO NPs were included intentionally to provide a baseline reference and to clearly illustrate the influence of nanoparticle incorporation. In addition, the membranes incorporated either graphite (**M1**, **M3**, **M5**, **M7**, **M9**, **M11**, and **M13**) or silver sulfide (**M2**, **M4**, **M6**, **M8**, **M10**, **M12**, and **M14**) as solid-contact charge-transfer materials. As shown in Figure 2, membranes **M1** and **M2** prepared without ZnO NPs exhibited noticeably reduced slopes. A similarly suppressed response was observed for membranes containing 0.1–0.4% (*w*/*w*) ZnO NPs (**M3**–**M10**) and due to their insufficient electrochemical performance, these compositions were excluded from further discussion.

Membranes containing graphite (**M11** and **M13**) as the charge-transfer material exhibited high responsiveness, with potential readings stabilizing within 3–7 s. In contrast, membranes containing silver sulfide (**M12** and **M14**) required 10–15 s to reach stable potential. The linear concentration range also differed slightly between the formulations, for **M11** and **M12**, it spanned 9.77 × 10^−5^ mol L^−1^ and 1.00 × 10^−2^ mol L^−1^ while for **M13** and **M14**, the range extended from 4.88 × 10^−5^ mol L^−1^ and 1.00 × 10^−2^ mol L^−1^. These results indicate faster response times for graphite-containing membranes, while both materials provided comparable concentration coverage. The faster response observed for graphite-containing membranes can be attributed to the superior electronic conductivity of graphite, which facilitates more efficient charge transfer at the membrane/electrode interface. In contrast, silver sulfide, while providing stable solid-contact behaviour, exhibits lower electronic mobility, leading to slightly slower potential stabilization. Additionally, the intimate contact between graphite particles and the ion-selective membrane likely enhances capacitive coupling, reducing the time required to reach equilibrium. These observations are consistent with previous reports highlighting that carbon-based materials often provide rapid response and low potential drift in solid-contact ISEs [35,36].

The limit of detection (LOD) and limit of quantification (LOQ) values for each electrode were calculated based on five repeated measurements. For each measurement, the mean and standard deviation were determined. The slope of the calibration curve was obtained using the LINEST function in Excel, and LOD and LOQ were then calculated using the following Equations (3) and (4):

(3) $$LOD=\;3.3\;*\;\frac{lowest\;meassured\;[K^{+}]}{slope}\;$$

(4) $$LOQ=10\;*\;\frac{lowest\;meassured\;[K^{+}]}{slope}$$

The reported values in Table 2 are presented as mean ± standard deviation, provide quantitative insight into the sensitivity of the electrodes and allow comparison across different compositions.

Membranes containing graphite (**M11** and **M13**) not only exhibited faster response times but also showed smaller error bars compared to their silver sulfide counterparts (**M12** and **M14**), indicating higher reproducibility and more stable potential readings. Among all tested compositions, the membrane containing 0.6% (*w*/*w*) ZnO NPs (**M13**) demonstrated the best performance, achieving a slope of 59.178 mV per decade with a correlation factor of 0.9989. This near-Nernstian behaviour highlights the optimal ion-to-electron transduction facilitated by the combined effect of the graphite solid-contact material and the appropriate ZnO NP content.

The enhancement in electrode slope and stability in the presence of ZnO nanoparticles can be attributed to their semiconductor nature and their ability to facilitate mixed ionic-electronic transport within the composite membrane. At the nanoscale, ZnO behaves as an *n*-type semiconductor, providing additional electronic conduction pathways that are not present in macroscopic ZnO. In our membrane, ZnO nanoparticles are dispersed within the ABS polymer matrix together with graphite, which already acts as a conductive additive. The interfacial contact between ZnO and graphite forms a percolation-type electronic network that lowers the overall charge-transfer resistance. This improves the electron-exchange kinetics between the ISE membrane and the underlying conductor. The ionic component of the transport is governed by the presence of KB(Ph)_4_, which serves as a lipophilic ionic site. The improved electronic conductivity provided by ZnO/graphite coupling ensures more efficient charge compensation for ionic fluxes associated with K^+^ recognition at the membrane/solution interface. In this way, ZnO nanoparticles act as a mediator that couples electronic transport (ZnO-graphite network) with ionic transport KB(Ph)_4_ within the ABS matrix), resulting in a more stable phase boundary potential and improved Nernstian response. Although a complete mechanistic description at the molecular level is beyond the scope of this work, the observed behaviour is consistent with previously reported semiconductor-enhanced ion-to-electron transduction in solid-contact ISEs, including the studies previously published by our research group [37,38].

### 3.2. Statistical Test of the K^+^ ISE

A one-way ANOVA test (*p* < 0.05) was performed using Microsoft Excel^®^ to assess the statistical significance of differences in response between membranes containing ZnO NPs and those without. The results are summarized in Table 3.

The calculated *F*-value exceeds the *critical F*-value, indicating that the differences are statistically significant. This confirms that the incorporation of ZnO NPs has a meaningful impact on the membrane response. The statistical analysis provides quantitative support for the observed enhancement in electrode performance, highlighting the role of ZnO as an effective functional additive in improving the electrochemical behaviour of the K^+^ ISE.

### 3.3. Selectivity of the K^+^ ISE

The selectivity of the K^+^ ISE is essential for accurate determination of potassium in the presence of potential interferants. Selectivity coefficients (log *K*^pot^) were determined using a fixed interference method, in which the electrode response to potassium ions was measured in the presence of a constant concentration (0.1 M) of the interfering ions and calculated according to Equation (2). This approach is recommended for potentiometric selectivity evaluation, as it effectively characterizes the discrimination of the primary ion over potential interferents [33]. The obtained selectivity coefficients, summarized in Table 4, are in good agreement with values reported in the literature [39]. Overall, the **M13** electrode demonstrates high selectivity for K^+^ over common interfering cations, highlighting its reliable performance for potassium detection.

#### Simulated Mixed-Ion Test

**Figure 3:**
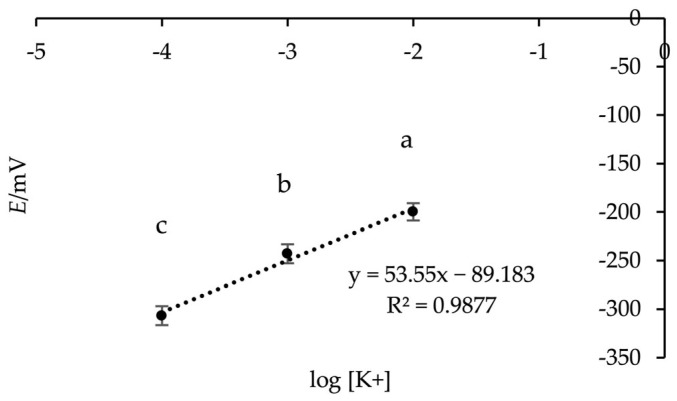
Response of the **M13** K^+^ ISE in mixed-ion solution at three different concentrations. Each data point corresponds to one of the three mixed-ion solutions: (a) 0.01 M KNO_3_, 0.1 M NaNO_3_ and 0.1 M NH_4_Cl; (b) 0.001 M KNO_3_, 0.01 M NaNO_3_ and 0.01 M NH_4_Cl; (c) 0.0001 M KNO_3_, 0.001 M NaNO_3_ and 0.001 M NH_4_Cl.

To evaluate the potentiometric response of the **M13** membrane under multi-ion conditions, three mixed-ion solutions containing K^+^, Na^+^ and NH_4_^+^ were prepared. The electrode was first conditioned overnight in 0.1 M KNO_3_ to ensure a stable baseline potential. EMF measurements were performed in three mixed solutions with the following compositions: (a) 0.01 M KNO_3_, 0.1 M NaNO_3_ and 0.1 M NH_4_Cl; (b) 0.001 M KNO_3_, 0.01 M NaNO_3_ and 0.01 M NH_4_Cl; and (c) 0.0001 M KNO_3_, 0.001 M NaNO_3_ and 0.001 M NH_4_Cl. As seen from Figure 3, the electrode exhibited a near-Nernstian slope of 53.6 mV/decade in the mixed-ion solution, slightly lower than the 59.17 mV/decade observed in pure K^+^ solutions. This minor decrease is attributed to competitive interference from Na^+^ and NH_4_^+^ ions. Overall, the electrode maintains a linear response and demonstrates good selectivity under simulated complex ionic conditions, confirming its robustness for K^+^ determination in multi-ion environments.

### 3.4. Dynamic Response of the K^+^ ISE

**Figure 4:**
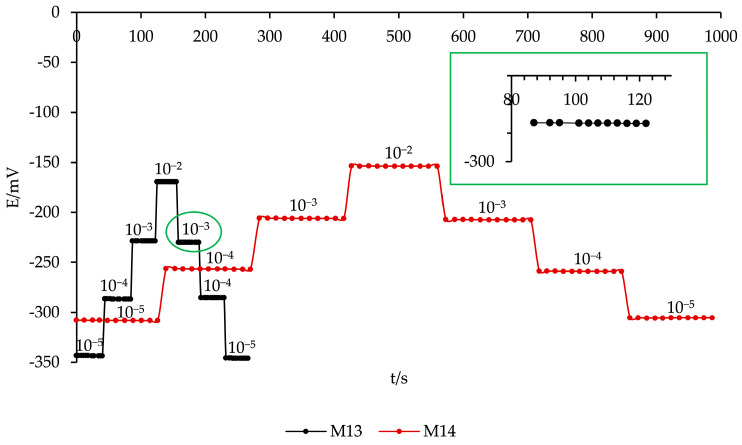
Dynamic response of **M13** and **M14** ISEs to stepwise K^+^ concentration changes. The main plot shows the full response, with the inset zooming (in green) in on the 10^−3^ M K^+^ step for the **M13** membrane to highlight stabilization kinetics.

The dynamic response of the K^+^ ISEs (**M13** and **M14**) was evaluated by monitoring the potential changes upon stepwise variations of the potassium concentration, ranging from 10^−5^ M to 10^−2^ M and then back to 10^−5^ M. As shown in Figure 4, the **M13** electrode, containing a graphite-based conductive additive, exhibited a faster response time of 3 to 7 s, whereas the **M14** electrode, incorporating Ag_2_S, responded more slowly, with a response time of 10 to 15 s. The faster response of **M13** can be attributed to the higher electronic conductivity of graphite compared to Ag_2_S, which facilitates quicker ion-to-electron transduction at the electrode interface. Both electrodes demonstrated fully reversible potential changes, indicating stable and reproducible performance. These results emphasize the significant role of the conductive additive type in determining response kinetics and confirm that **M13** is particularly suitable for rapid potassium measurements under dynamic conditions.

### 3.5. Performance Benchmarking

To evaluate the performance of the **M13** K^+^ ISE in the context of recent literature, key analytical parameters were compared, including Nernstian slope, LDP, LOD, selectivity toward common interfering ion (Na^+^), response time and lifetime. Table 5 summarizes the comparison with selected recent reports.

The **M13** electrode exhibit near-Nernstian slopes and competitive detection limits, consistent with established polymeric K^+^ ISE. The overall analytical behaviour of our electrode aligns well with typical near-Nernstian performance, demonstrating their reliability and practical applicability.

### 3.6. Characterisation of the K^+^ ISE

**Figure 5:**
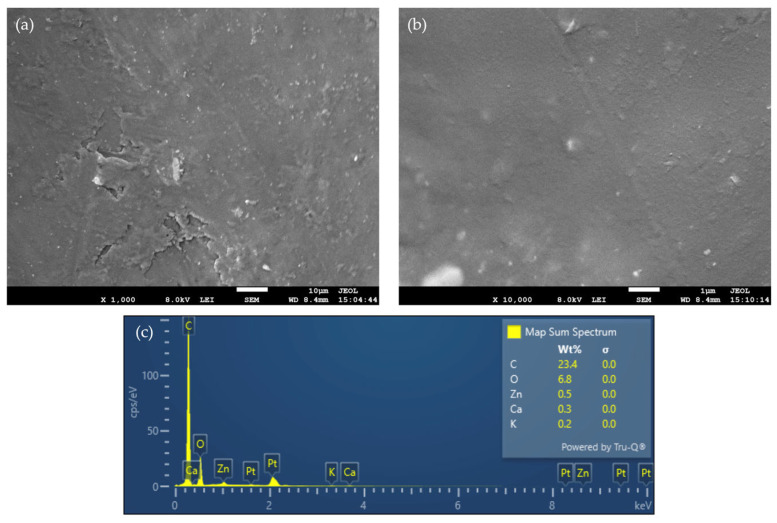
SEM image of **M13** membrane at (**a**) 1000× and (**b**) 10,000× magnifications; (**c**) EDS spectrum of the membrane surface showing elemental composition. The prominent Pt peaks originate from the 5 nm sputter coating applied to the sample.

The morphology and elemental composition of the **M13** membrane were investigated by SEM, EDS, XRF and ATR-FTIR analyses. SEM micrographs recorded at 1000× and 10,000× magnifications (Figure 5a,b) show a homogeneous membrane surface with well-dispersed ZnO NPs throughout the polymeric matrix. Higher-magnification images provide clearer insight into the surface texture and nanoparticle distribution, confirming the uniform incorporation of ZnO NPs. EDS analysis (Figure 5c) further confirms the presence of Zn and O, alongside elements from the polymeric matrix, verifying the elemental composition at the membrane surface. The prominent platinum peaks originate from the 5 nm sputter coating applied prior to SEM imaging. The Zn signal, measured at approximately 0.5 wt%, is close to the detection limit of standard EDS, and thus the elemental quantification should be considered semi-quantitative. Additional SEM images at 20,000× and 50,000× magnifications, as well as the complete EDS analysis, are provided in the Supplementary Information.

**Figure 6:**
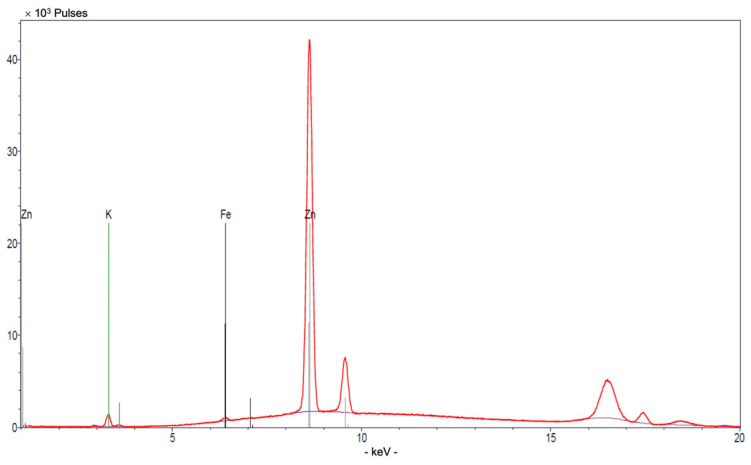
XRF spectrum of **M13** membrane.

XRF analysis (Figure 6) shows a clear and intense Zn signal, confirming the presence of ZnO NPs within the membrane bulk.

**Figure 7:**
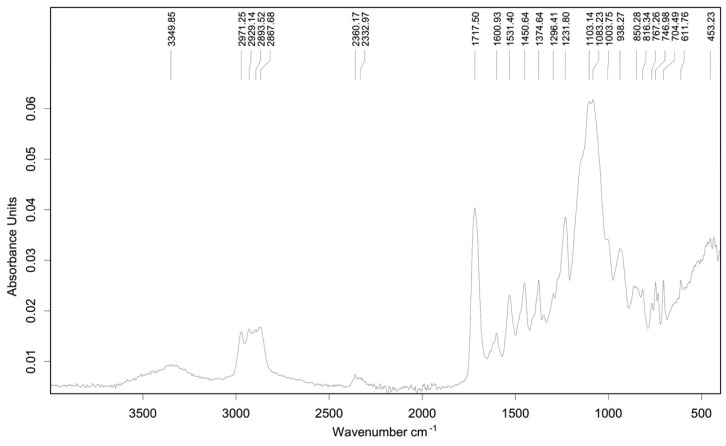
ATR-FTIR spectrum of **M13** membrane.

The ATR-FTIR spectrum of the **M13** membrane (Figure 7) is dominated by the characteristic bands of ABS, the main component of the membrane (95.4 wt%). The peaks at 2971, 2921, 2883 and 2857 cm^−1^ correspond to aliphatic C-H stretching vibrations of the polymer backbone. A band at ~1600 cm^−1^ is assigned to aromatic C=C stretching of the styrene units, while the bands at 1451 and 1374 cm^−1^ are related to CH_2_ and CH_3_ deformation vibrations. Additional bands in the 1296–1003 cm^−1^ region arise from C-H bending and aromatic ring vibrations. Aromatic modes originating from potassium tetraphenylborate likely overlap with these bands due to the presence of phenyl groups. Bands observed between 611 and 453 cm^−1^ are attributed to Zn-O stretching vibrations, confirming the presence of ZnO nanoparticles. A broad band around 3349 cm^−1^ is assigned to O-H stretching from adsorbed moisture or surface hydroxyl groups on ZnO. The contribution of graphite is not clearly distinguishable due to its low infrared activity and low concentration.

Overall, the combined SEM, EDS, XRF, and ATR-FTIR analyses provide consistent morphological and compositional evidence for the successful incorporation of ZnO nanoparticles into the polymeric membrane, supporting the link between ZnO incorporation and the observed potentiometric response.

## 4. Conclusions

In this study, we successfully fabricated a K^+^ selective ISE using 3D printing with a simplified and innovative membrane composition. The ISE, composed of potassium tetraphenylborate, graphite, and industrial ABS (3:1:95.4 mass ratio) with 0.6% ZnO NPs, exhibited near-Nernstian behaviour (slope: 59.178 mV per decade), a low limit of detection (2.06 × 10^−5^ mol L^−1^), and high selectivity against common interfering ions. SEM, EDS, XRF and ATR-FTIR analyses confirm uniform ZnO incorporation, supporting its role in enhancing the potentiometric response, while graphite ensures fast and reproducible performance compared to silver sulfide. These results demonstrate the potential of combining innovative membrane materials with 3D printing to develop reliable and selective potassium sensors.

## Figures and Tables

**Table 1:** Composition of membranes with KB(Ph)_4_, graphite or Ag_2_S, industrial ABS and ZnO NPs in different mass ratios, prepared for testing the response of potassium ions.

Membrane	KB(Ph)_4_ %	Graphite %	Ag_2_S %	Ind. ABS %	ZnO NPs %
**M1**	3	1		96	
**M2**	3		2	95	
**M3**	3	1		95.9	0.1
**M4**	3		2	94.9	0.1
**M5**	3	1		95.8	0.2
**M6**	3		2	94.8	0.2
**M7**	3	1		95.7	0.3
**M8**	3		2	94.7	0.3
**M9**	3	1		95.6	0.4
**M10**	3		2	94.6	0.4
**M11**	3	1		95.5	0.5
**M12**	3		2	94.5	0.5
**M13**	3	1		95.4	0.6
**M14**	3		2	94.4	0.6

**Table 2:** LOD and LOQ for the membranes with ZnO NPs (**M11**–**M14**) and without (**M1** and **M2**).

Membrane	LOD	LOQ
**M1**	7.11 × 10^−5^ ± 0.18	1.4 × 10^−4^ ± 0.53
**M2**	3.79 × 10^−5^ ± 0.44	1.13 × 10^−4^ ± 1.35
**M11**	4.00 × 10^−5^ ± 0.26	7.28 × 10^−5^ ± 0.80
**M12**	3.45 × 10^−5^ ± 0.09	6.93 × 10^−5^ ± 0.28
**M13**	2.06 × 10^−5^ ± 0.15	3.67 × 10^−5^ ± 0.46
**M14**	1.59 × 10^−5^ ± 0.14	3.37 × 10^−5^ ± 0.41

**Table 3:** Analysis of variance (ANOVA) evaluating the statistical significance of differences between the membranes.

Source of Variation	Sum of Squares	Degrees of Freedom	F	*p*	F (Critical)
Between groups	186,890.485	5	14.23184	3.54 × 10^−9^	2.36827
Within groups	157,582.332	60			
Total	344,472.817	65			

**Table 4:** Selectivity coefficients for biologically relevant interfering cations for M13 membrane.

Interferent (B)	Concentration/M	${logK}_{K^{+},B}^{pot}$
Sodium	0.1	−3.12
Calcium	0.1	−3.22
Ammonium	0.1	−1.98
Magnesium	0.1	−3.48

**Table 5:** Comparison of key analytical parameters of the **M13** K^+^ ISE with recent literature.

Ion-to-Charge Transducer	Slope (mV/decade)	LDP (mol L^−1^)	LOD (mol L^−1^)	Selectivity (log K^+^/Na^+^)	Response Time (s)	Lifetime	Ref.
Graphite	59.178	4.88 × 10^−5^–10^−2^	2.06 × 10^−5^	−3.12	3–7	>1 year	This work
Mesoporous carbon black	56.6	10^−5^–10^−2^	1.9 × 10^−6^	−3.72	~20	3 weeks	[40]
Carbon black	56.8	10^−4^–10^−1^	10^−4^	/	4	/	[41]
Carbon black	59.2	10^−4^–10^−1^	10^−5^	/	4	/	[8]
ZnO NPs	56.07	10^−5^–10^−1^	3.66 × 10^−6^	/	4–8	>5 months	[42]

## Data Availability

The original contributions presented in this study are included in the article/Supplementary Material. Further inquiries can be directed to the corresponding author.

## Supplementary Materials

The following supporting information can be downloaded at: https://www.mdpi.com/article/10.3390/s26061960/s1, Figure S1: SEM image of **M13** membrane at 20,000× magnifications. Figure S2: SEM image of **M13** membrane at 50,000× magnifications. Figure S3: SEM image of **M13** membrane at 1000× magnifications. Figure S4: EDS analysis of **M13** membrane: quantitative elemental composition of the sample (top) and corresponding individual elemental distribution maps (bottom).

## Abbreviations

The following abbreviations are used in this manuscript:

ABS	Acrylonitrile Butadiene Styrene
Ag_2_S	Silver Sulfide
Ag/AgCl	Silver/Silver Chloride
ANOVA	Analysis of Variance
ATR-FTIR	Attenuated total reflectance Fourier transform infrared spectroscopy
Ca(NO_3_)_2_ × 4H_2_O	Calcium Nitrate Tetrahydrate
EDS	Energy-dispersive X-ray spectroscopy
EMF	Electromotive Force
ISE	Ion-Selective Electrode
KB(Ph)_4_	Potassium Tetraphenyl Borate
KNO_3_	Potassium Nitrate
LOD	Limit of Detection
LOQ	Limit of Quantification
Mg(NO_3_)_2_ × 6H_2_O	Magnesium Nitrate Hexahydrate
NaNO_3_	Sodium Nitrate
NH_4_Cl	Ammonium Chloride
NPs	Nanoparticles
OD-MWCNT	Octadecylamine-Functionalized Multi-Walled Carbon Nanotube
PEDOT	Poly(3,4-ethylenedioxythiophene)
POT	Poly(3-octylthiophene-2,5-diyl)
R^2^	Factor of Correlation
SEM	Scanning electron microscopy
ZnO	Zink Oxide
XRF	X-ray fluorescence
