# Supplementary material for: 3D Printed Ion-Selective Electrodes Enriched with ZnO Nanoparticles for Potassium Detection
Source: Sensors (Basel). 2026 Mar 20;26(6):1960. doi: 10.3390/s26061960 (PMC13030318; doi:10.3390/s26061960)
Supplement: Supplementary file 1 [file sensors-26-01960-s001.zip › sensors-4175451-supplementary.pdf]

Supplementary Information

3D Printed Ion-Selective Electrodes Enriched with ZnO Nanoparticles for Potassium Detection

Ita Hajdin [1] and Ante Prkić [1,]*

[1] Faculty of Chemistry and Technology, University of Split, Ruđera Boškovića 35, 21000 Split, Croatia.; ihajdin@ktf-split.hr; ante.prkic@ktf-split.hr

* Correspondence: ante.prkic@ktf-split.hr; Tel.: +385 21-329-462

1. SEM

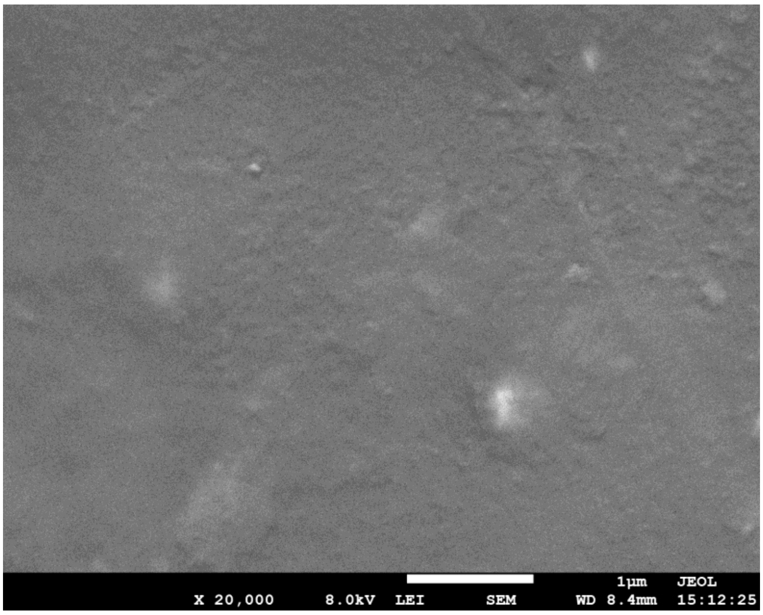

Figure S1. SEM image of **M13** membrane at 20,000× magnifications.

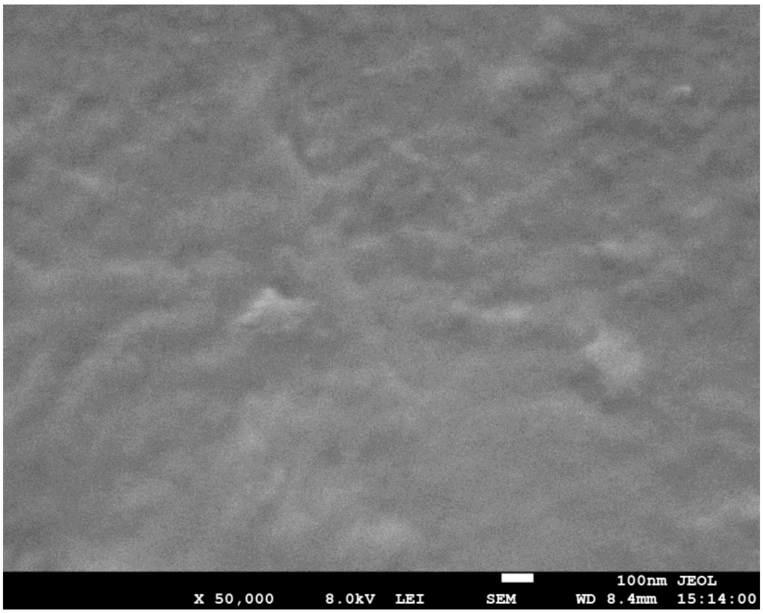

Figure S2. SEM image of **M13** membrane at 50,000× magnifications.

2. EDS

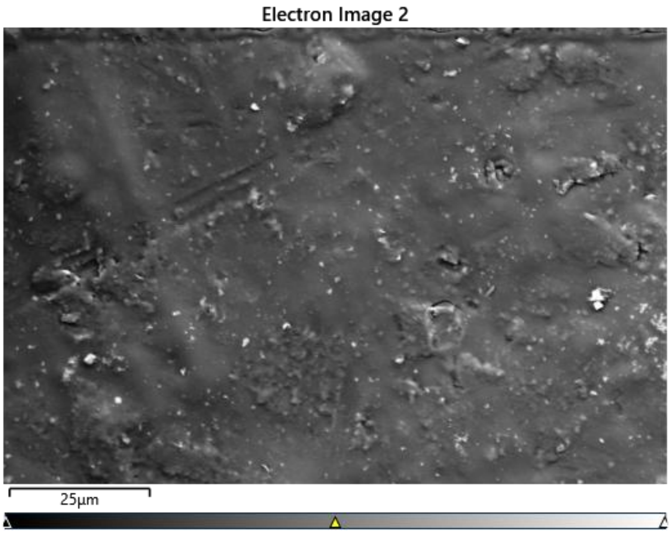

Figure S3. SEM image of **M13** membrane at 1000× magnifications.

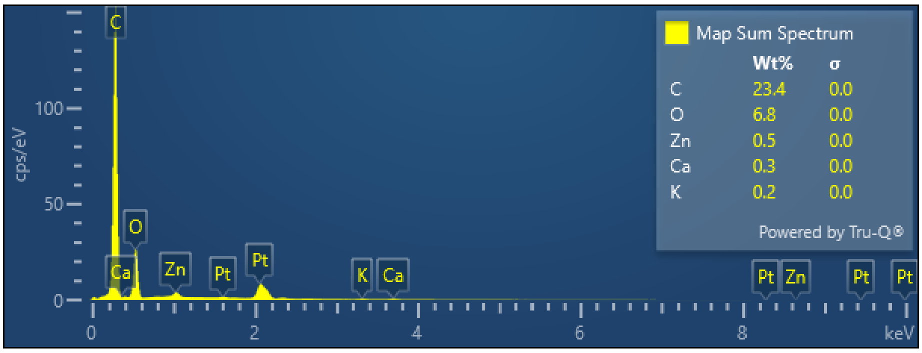

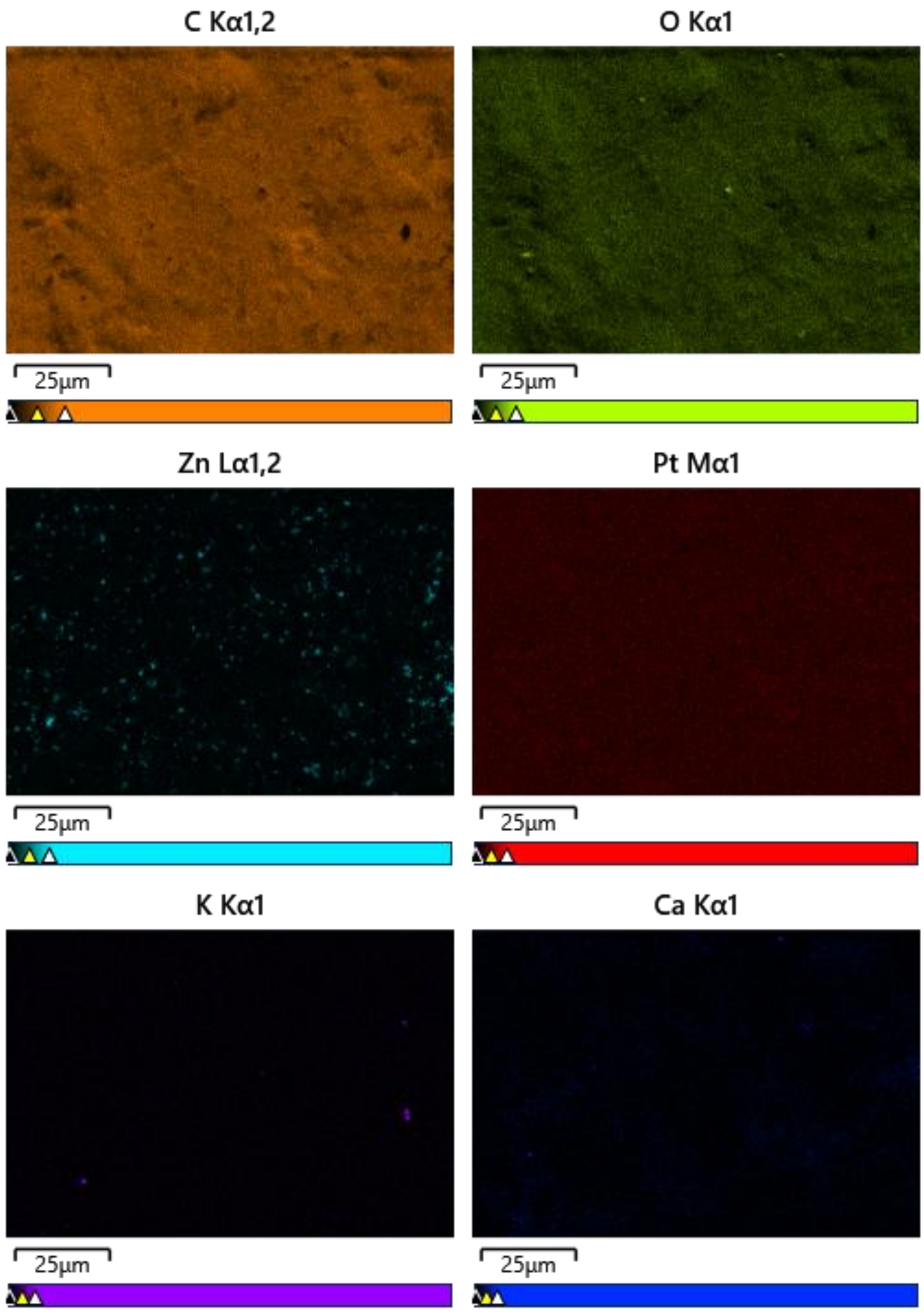

Figure S4. EDS analysis of **M13** membrane: quantitative elemental composition of the sample (top) and corresponding individual elemental distribution maps (bottom).
